# Adapted Physical Activity for Breast Cancer Patients Treated with Neoadjuvant Chemotherapy and Trastuzumab Against HER2 (APACAN2): A Protocol for a Feasibility Study

**DOI:** 10.3389/fonc.2021.744609

**Published:** 2021-12-13

**Authors:** Angeline Ginzac, Maureen Bernadach, Ioana Molnar, Martine Duclos, Emilie Thivat, Xavier Durando

**Affiliations:** ^1^ INSERM U1240 IMoST, Université Clermont Auvergne, Clermont-Ferrand, France; ^2^ Département de Recherche Clinique, Délégation Recherche Clinique et Innovation, Centre Jean PERRIN, Clermont-Ferrand, France; ^3^ Centre d’Investigation Clinique UMR501, Clermont-Ferrand, France; ^4^ Service d’Oncologie Médicale, Centre Jean PERRIN, Clermont-Ferrand, France; ^5^ CHU Clermont-Ferrand, Service de Médecine du Sport et des Explorations Fonctionnelles, Université Clermont Auvergne, INRAE, UNH, Clermont–Ferrand, France

**Keywords:** HER2-positive breast cancer, adapted physical activity, neoadjuvant chemotherapy, HER2-directed therapy, feasibility study

## Abstract

**Background:**

The standard care for HER2-positive breast cancer is chemotherapy plus a HER2-directed therapy. This can lead to treatment-induced cardiotoxicity. On the other hand, the practice of physical activity is known to improve cardiac function; thus HER2-positive breast cancer patients could draw particular benefit from physical activity during treatment. However, at the time of diagnosis for breast cancer, the majority of patients are insufficiently active according to physical activity recommendations of World Health Organisation, and it is difficult to remain or become active during the treatment. There is a lack of data in the literature on the optimal program to propose to patients to encourage them to be active during treatment. The aim of our study is to assess the feasibility of a home-based physical activity program during neoadjuvant chemotherapy and trastuzumab for HER2-positive breast cancer.

**Methods:**

The APACAN2 study is a single-centre, non-randomized interventional trial. Patients with HER2-positive breast cancer treated with anthracycline-based neoadjuvant chemotherapy and trastuzumab are eligible for enrolment. The supervised home-based physical activity program takes place during neoadjuvant chemotherapy (NACT). It combines aerobic and strengthening exercises. The primary endpoint is the proportion of patients reaching the international physical activity recommendations, i.e. 150 minutes of moderate-intensity activity per week at the end of NACT. The study started in April 2018 and seventy patients are expected to be recruited.

**Discussion:**

In the literature, the majority of studies on practice of physical activity in breast cancer focus on adjuvant chemotherapy or on the period after the end of treatment. To the best of our knowledge, the APACAN2 study is the first to evaluate a home-based physical activity program during neoadjuvant chemotherapy for HER2-positive breast cancer.

**Trial Registration Number:**

Clinicaltrials.gov: NCT02963363, registered on July 11, 2016. Identifier with the French National Agency for the Safety of Medicines and Health Products N°ID RCB 2016-A01344-47, registered in August 2016. Protocol: version 8, 24 February 2021.

## 1 Introduction

Worldwide, breast cancer is the most commonly diagnosed cancer among women and is also the leading cause of cancer death (1). Overexpression of human epidermal growth factor receptor 2 (HER2) concerns approximately 15% of breast cancer patients. This subtype of cancer is associated with a poorer disease-free survival and overall survival (2, 3). The standard care to treat these tumours is chemotherapy plus HER2-directed therapy administered in combination with standard chemotherapy (anthracycline and taxane-based). This treatment reduces both the rate of recurrence and the mortality rate, respectively by half and by one third. However, trastuzumab (the most commonly used HER2-directed therapy) is known to be associated with cardiotoxicity (4). Anthracycline-based chemotherapy also causes cardiotoxicity (5, 6). Between 1 and 4% of patients treated with trastuzumab develop heart failure is and nearly 10% have asymptomatic LVEF reduction (7–10). It has been estimated that the cumulative incidence rate for major cardiac events (as for example pulmonary edema, cardiomyopathy; cardiovascular death) is 6.6% for sequential therapy, i.e. anthracycline and trastuzumab (11).

As a result, HER2-positive breast cancer patients are exposed to treatment-induced cardiotoxicity. This can have serious consequences, such as treatment discontinuation (12).

Physical exercise is known to improve quality of life during and after breast cancer treatment (13–16). It can also be beneficial for cardiac function. Eight weeks of aerobic practice during adjuvant chemotherapy for breast cancer have led to an increase in oxygen peak consumption reflecting an improvement in cardio-respiratory capacities (17). The longer the physical activity program, the greater the improvement in oxygen peak consumption (ranging from 0.6 ml/min/kg after 12 weeks to 1.83 ml/min/kg after 27 weeks of practice) (18–20). In addition, physical activity could protect the cardiovascular system, so that it could be a strategy to limit treatment-induced cardiotoxicity in the HER2-positive population. However, at diagnosis of breast cancer, the majority of patients have insufficient levels of physical activity and do not reach the international recommendations (21–25). These recommendations are issued by the World Health Organisation (WHO) for the healthy population, and correspond to 150 minutes of moderate-intensity activity or 75 minutes of vigorous activity or an equivalent combination of moderate and vigorous activity per week (26) and to limit time spent in sedentary behaviors. Nevertheless, it can be difficult to maintain physical activity during treatment because of several barriers relating to care, and also personal barriers (27).

Numerous interventional clinical trials have been set up to promote adapted physical activity during breast cancer treatment. The notion of adapted physical activity refers to physical activities and sports that are adapted to the capacities of people because of their health condition. The proposed programs are heterogeneous in terms of exercises (aerobic and/or muscular strengthening), duration of sessions, duration of the program (a few weeks to several months), intensity or even the mode of practice (alone or in a group, supervised or not) (27).

In the literature data, there are few trials exploring the interest in physical activity for the HER2-positive breast cancer subpopulation. Haykowski’s study (aerobic exercise, three sessions of 30 to 60 minutes/week) concerned the first four months of trastuzumab administration and did not evidence any improvement in cardiac function (28). On the contrary, left ventricular cavity dilation and significant decreases in the ventricular ejection fraction were observed. The authors pointed out that the poor adherence to exercise sessions could explain this result, because cardiopulmonary function improved for patients that completed ≥ 55% of the sessions. A randomized controlled study among HER2-positive patients showed that patients who followed the aerobic intervention during their neoadjuvant chemotherapy (3 sessions per week, one-to-one supervised sessions) improved their cardiopulmonary function while it decreased in the control group patients (29). In France, the CARDAPAC phase II study conducted on HER2-positive patients treated with trastuzumab alone has just been completed (30). The aim was to assess the impact of 3 months’ aerobic exercise on cardiac function and on the incidence of cardiotoxicity. The program started at the end of chemotherapy and consisted in 3 sessions of 45 minutes a week.

The practice of physical activity is recommended as soon as possible at the beginning of treatment for breast cancer patients (31–33). However, in the literature, the majority of the programs are offered in the course of adjuvant chemotherapy or in the post-treatment period. Furthermore, there is a lack of information in the literature about the interest of physical activity practice for HER2-positive breast cancer patients. In this context, Jean PERRIN Centre has initiated a prospective interventional study in order to study the feasibility of a home-based physical activity intervention among HER2-positive breast cancer patients currently treated with neoadjuvant chemotherapy + targeted therapy. The objective of the intervention is to achieve or maintain a physical activity level corresponding to the WHO international recommendations at the end of chemotherapy and to limit time spent in sedentary behaviors.

## 2 Methods and Analysis

### 2.1 Study Design

APACAN2 is a French single-centre, prospective, interventional, non-randomized trial designed to assess the feasibility of a home-based adapted physical activity (APA) intervention during neoadjuvant chemotherapy for early HER2-positive breast cancer (NCT02963363).

Seventy patients are expected to be recruited. Patient enrolment is expected to take 6 years and the study duration for each patient is 20 months.

Participants can withdraw at any time. Data obtained will be retained with consent, and any reasons given for withdrawal will be recorded.

### 2.2 Coordination

The Centre Jean PERRIN is the sponsor and is responsible for coordination, trial management, data management and trial monitoring.

### 2.3 Study Objectives and Endpoints

#### 2.3.1 Primary Objective and Endpoint

The primary objective of the APACAN2 trial is to demonstrate the feasibility of a home-based adapted physical activity (APA) program for patients with HER2 positive breast cancer receiving neoadjuvant chemotherapy. Therefore, the primary endpoint is the proportion of patients reaching the international recommendations for physical activity at the end of chemotherapy. It will be evaluated using the recent physical activity questionnaire (RPAQ) which will be completed before and at the end of neoadjuvant chemotherapy (34).

#### 2.3.2 Secondary Objectives and Endpoints

The secondary objectives of the APACAN 2 trial are:

- assessment of the impact of the APA program on exercising time and sedentary time; quality of life; fatigue; weight; physical capacities; physical activity in the rest of the day; lipid profile; ventricular ejection fraction. This will be evaluated using questionnaires [RPAQ (34), QLQ-C30 (35), MFI-20 (36)], physical tests, blood tests and cardiac ultrasound. This data will be collected at baseline, at the end of chemotherapy and at the beginning and the end of targeted therapy.
- description of the longitudinal evolution of physical activity and sedentary behaviour at each step of treatment,
- exploration of barriers to program uptake,
- conditions of the return to work,
- exploration of changes in cancer treatment.

### 2.4 Study Procedures and Participant Timeline

An overview of the study assessments and procedures is presented in **Table 1**.

**Table 1:** Study timeline.

	Inclusion (before NACT)	Standard evaluation during NACT	End of NACT +/- trastuzumab	First administration of adjuvant trastuzumab	Last administration of adjuvant trastuzumab
		APA intervention			
Consent	✓				
Clinical examination	✓	✓	✓	✓	✓
Previous and ongoing treatments	✓	✓	✓	✓	✓
Toxicities assessment	✓	✓	✓	✓	✓
Follow-up of cancer treatment (adaptation, delay, termination)		✓	✓	✓	✓
Anthropometric measures	✓	✓	✓	✓	✓
Physical capacities evaluation					
Measurement of cardiorespiratory aerobic capacity	✓				✓
Measurement of muscle strength and muscular endurance	✓		✓	✓	✓
Physical activity and sedentarity (modified RPAQ)	✓		✓	✓	✓
QLQ-C30 and MFI20 questionnaire	✓		✓	✓	✓
Lipid test	✓		✓	✓	✓
Cardiac ultrasound	✓		✓	✓	✓
					

APA, Adapted physical activity.

NACT, Neoadjuvant chemotherapy.

RPAQ, Recent Physical Activity Questionnaire.

QLQ-C30, Quality of Life Questionnaire - Core 30.

MFI-20, Mutidimensional Fatigue Inventory.

**Figure 1:**
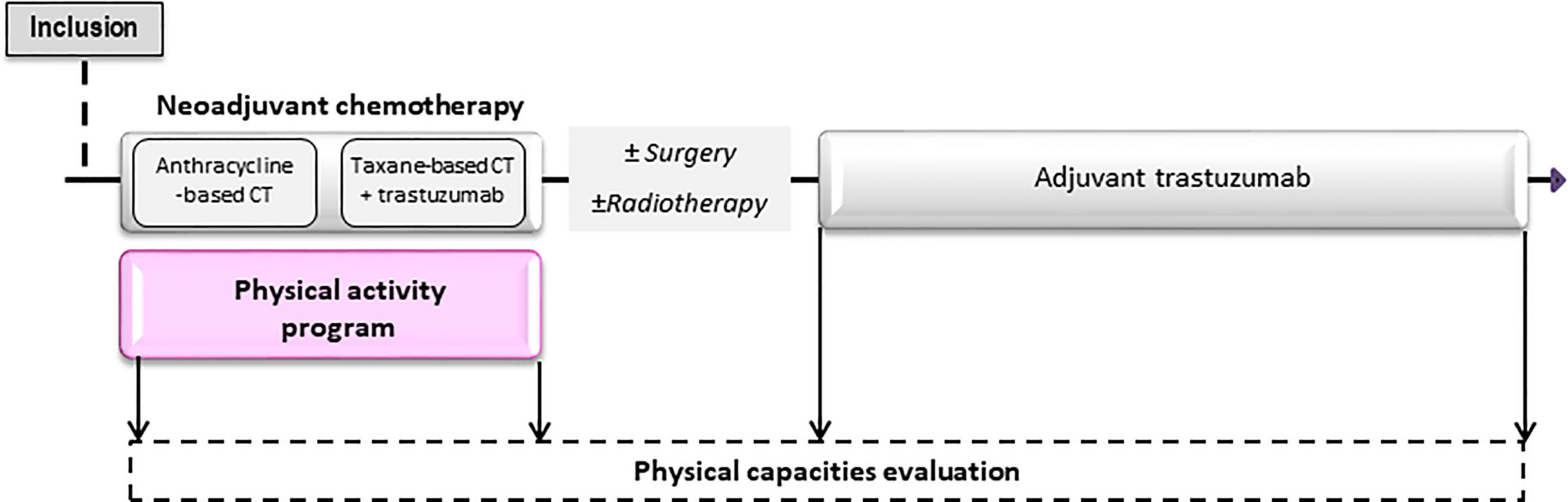
Design of the APACAN2 study. The APA program take place from the beginning to the end of neo-adjuvant chemotherapy. Four follow-up visits are planned during the study.

Four visits are follow-up appointments for each enrolled patients: inclusion [before the beginning of neoadjuvant chemotherapy (NACT)], at the end of NACT, at the beginning and at the end of HER2-targeted therapy. The study layout is presented in **Figure 1**.

### 2.5 Measures at Each of the Four Assessments

#### 2.5.1 Physical Activity and Sedentariness

The assessment of physical activity and sedentariness will be performed using the modified RPAQ. This questionnaire covers four areas of activity: domestic activities, leisure activities, professional activities, and modes of travel. This questionnaire provides information on the time spent on different activities in each of the four areas mentioned above. Specific energy expenditure is associated with each physical activity, which makes it possible to estimate the overall energy expenditure of patients using Ainsworth compendium (37). It also provides information on the time spent sedentary (work, leisure, travel).

#### 2.5.2 Measurement of Muscle Strength and Muscular Endurance

An isokinetic dynamometer will be used to measure the maximum knee extension torque. Subjects will perform the movement with their non-dominant leg (which will be determined as the leg not used to kick a ball). Measurements will be made at different speeds (30, 60 and 120°/s). For each speed, two trials of 3 successive repetitions will be performed and the best performance will be kept as the maximum isokinetic torque at a given speed. The subjects will have 2 minutes of rest between each trial. Finally, the maximum isometric knee extension torque will be measured at a 120° angle. Subjects will perform a maximal contraction over 5 seconds or until the isometric torque trace peaks. Subjects will be given 3 trials and 3 minutes of rest between each trial. The best trial will be considered the maximum isometric knee extension torque.

For the upper limbs, muscular strength will be measured with both right and left arm using a dynamometer (hand grip Takei TK 200). Measures will be repeated three times and the best score for each side is retained.

To measure muscular endurance of the lower limbs, the objective for the patient is to maintain a level of force corresponding to 75% of her maximum voluntary force for as long as possible.

#### 2.5.3 Measurement of Cardiorespiratory Aerobic Capacity

This measure is not mandatory for all patients in order not to delay the beginning of treatment (not related to primary objective) and is expected to be performed twice during participation in the study: at baseline and at the end of the study.

The subjects performed a progressive cycling test on an electromagnetically braked cycle ergometer (Ergoline, Bitz, Germany) until volitional exhaustion to determine the maximal values of ventilation (VEmax), oxygen uptake (VO2max), carbon dioxide output and respiratory exchange ratio (RERmax) by direct method (Oxycon Pro, JAEGER, Germany). VO2 and VCO2 were measured breath-by-breath through a mask connected to O2 and CO analysers (Oxycon Pro-Delta, Jaeger, Hoechberg, Germany). Calibration of gases analysers was performed with commercial gases of known concentration. Ventilatory parameters were averaged every 30 s. Electrocardiogram and heart rate (HR) were measured continuously using 10 precordial electrodes. The first stage of the test lasted 3 min, and the initial power output was 35 W. Power output was then increased by 20 W every 2 min 30 s. Pedaling rate was maintained at 60 revolutions per minute. Criteria for the achievement of VO2max were subjective exhaustion the participants’ maximal HR (HRmax) was closed to their age-predicted maximum HR (i.e., 220-age ± 10 beats.min-1) and/or Respiratory Exchange ratio (RER, VCO2/VO2) above 1.02 and/or a plateau of VO2.

#### 2.5.4 6-Minute Walking Test

The test will be run twice in order to reduce the “first time” effect. This test measures the distance a patient can walk in six minutes and thus allows the evaluation of the global and integrated responses of all systems involved during exercise and is considered a proxy for cardiorespiratory capacity. A score is obtained in meters. This test will be performed following the guidelines of the American Thoracic Society (38)and has been tested and validated in the past (39).

#### 2.5.5 Self-Administered Questionnaires

The QLQ-C30 (Quality of Life Questionnaire-Core 30) and the MFI20 (Multidimensional Fatigue Inventory) will enable us to evaluate respectively the patient’s quality of life (QoL) and fatigue during the study.

#### 2.5.6 Anthropometric Measures

Weight will be measured to within ± 0.1 kg on the same scales. The body mass index will be calculated from this data.

Waist circumference will be measured with a tape measure.

Pulse, blood pressure and temperature will be measured by a nurse at the investigating centre.

#### 2.5.7 Lipid Test

A lipid assessment will be performed at inclusion, at the end of neoadjuvant chemotherapy and on the day of the first and last administration of anti-HER2 therapy. This assessment will include the determination of total cholesterol, HDL cholesterol, LDL cholesterol and triglycerides. These parameters give an indication of possible cardiovascular risk factors. It will require the collection of 5 ml of blood.

#### 2.5.8 Cardiac Ultrasound

A cardiac ultrasound scan will be performed at each of the four appointments. The ventricular ejection fraction results will be evaluated by a cardiologist and compared in order to assess the effect of the APA program on the impact of treatments on cardiac capacity. The cardiac ultrasound is an essential measure for management of cardiovascular function. The same method will be used for each of the assessments for each patient.

### 2.6 Selection Criteria

The inclusion and non-inclusion criteria are presented in **Table 2**. Patients will be eligible for the study if they have HER2-positive breast cancer with an indication for treatment with neoadjuvant chemotherapy (anthracycline-based) and targeted therapy. They will be ineligible if they have a contraindication for physical activity, a metastatic cancer or a Karnofsky index ≤ 90%

**Table 2:** Selection criteria.

**Inclusion criteria**	- Woman 18 years old and older - Patient with HER2-positive breast cancer, histologically confirmed, eligible to a neoadjuvant chemotherapy and an anti-HER2 target therapy - Covered by social security system - Signed informed consent - Certificate of non-contraindication to the practice of physical activity
**Non-inclusion criteria**	- History of cancer in the last five years except basocellular - Metastatic cancer - Karnofsky index ≤ 90% - Men - Pregnant women - Patient with psychiatric or cognitive disorders - Patient deprived of liberty by judicial or administrative decision - Contraindication to the practice of physical activity - Insufficient knowledge or understanding of the French language to fill in self-questionnaires correctly or to answer an interrogation - Participation to another clinical study with a similar objective

### 2.7 Description of the APA Intervention

The intervention takes place during the neoadjuvant chemotherapy period, i.e. lasting about 18 weeks. The APA program is composed of aerobic exercises and muscle strengthening. An exercise booklet will explain to the patients the different exercises to perform.

Before the beginning of the program, the medical-sports educator (MSE) will demonstrate every exercise of the booklet to the patients. Thereafter patients will perform the program at home. The MSE will call each patient every week to review the program progress and increase or decrease the intensity of exercise according to the patient’s experiences.

The aerobic exercises consist in walking on at least five days a week. The duration of walking sessions should increase every week to reach 30 minutes walking per day.

Three times a week, the patients are to perform muscle strengthening with at least 4 different exercises, to mobilize the lower and upper limbs, among those proposed in the booklet. For each of them, the patient are to perform 3 sets of 8 to 12 repetitions with 1 minute of rest between each set.

Patients are asked note all the exercise sessions they do. They should also mention any other physical activity week by week, such as hiking, gardening or cleaning.

### 2.8 Statistical Analysis

The analysis will be done by the biostatistician of clinical research department in Jean PERRIN Center.

#### 2.8.1 Sample Size

Our hypothesis is that at least 65% of the patients will have reached the physical activity recommendations at the neoadjuvant post-chemotherapy evaluation. A total of 70 patients is required to have an accuracy of ± 10% with a 95% confidence interval (CI). Given this CI, the trial will be considered positive if more than 75% of our population complies with the international recommendations.

#### 2.8.2 Data Analysis

##### 2.8.2.1 Primary Analysis

The primary endpoint will be the percentage of individuals with physical activity after neoadjuvant chemotherapy, as assessed by the RPAQ with 150 minutes or more per week of moderate intensity or 75 minutes or more of vigorous intensity endurance activity or an equivalent combination of moderate to vigorous activity per week. The 95% confidence interval of this percentage will be calculated using Pearson’s approximation. If this confidence interval is greater than 65%, the intervention will be considered effective. Its feasibility will then be demonstrated. If it only reaches 65% but is greater than 50%, the intervention will be considered “questionable” and below this, its effectiveness will be considered nil.

##### 2.8.2.2 Secondary Analysis

To assess the impact of the APA program, a comparative analysis of these before/after changes will be carried out on physical activity (MET-h/week), sedentariness (minutes), QoL (overall score, sub scores), fatigue, weight, physical capacities (endurance, muscular strength, flexibility), lipid balance, and cardiac function. The following common tests will be used: paired Student’s t-test, Mann-Whitney U-test, and Chi² for paired series for qualitative criteria. We will also evaluate the impact of levels of physical activity and compliance with recommendations on these parameters using the following tests: ANOVA, Pearson’s correlation or Spearman rank tests depending on whether the distributions are Gaussian and homoscedastic.

The effect of the physical activity levels on compliance to the program will be tested by correlations. The Hryniuk score will be used to explore the treatment changes occurring.

Concerning the resumption of professional activity, a survival curve for time to recovery will be calculated.

Concerning the analysis of the longitudinal evolution of physical activity and time spent in sedentary behaviour, a mixed-model ANOVA will be used.

The evaluation of barriers to physical activity adherence will be carried out using the following tests: ANOVA, Pearson’s correlation or Spearman’s rank tests depending on whether the distributions are Gaussian and homoscedastic. A logistic regression will test the respective influence of the different factors on whether or not the recommendations are met.

The secondary endpoints will be compared to control data from the Jean Perrin Centre or with data from the literature whenever possible.

The standard significance level (p < 0.05) will be used for these analyses. As this trial is exploratory, no correction for multiple comparisons will be made.

### 2.9 Data Management And Monitoring

The data collected for the trial will be entered on an electronic case report form (eCRF) (EnnovClinical). The people with access to the data will be the investigators, the clinical research associates, the project leader and the biostatistician. They are authorized professionals and are subject to professional secrecy. The investigator will ensure the accuracy, completeness, and reliability of the data recorded (pseudonymized patient data) and the provision of answers to data queries.

Regular monitoring will be carried out by a clinical research associate mandated by the sponsor. The objectives will be to ensure the correct conduct of the study in each centre, the collection and recording of data generated in writing, its documentation, recording and reporting, in accordance with the legislative and regulatory provisions in force. Monitoring reports will ensure traceability.

### 2.10 Trial Status

The APACAN2 trial is currently recruiting. Participant recruitment began in April 2018 and is expected to finish in December 2022. The approved protocol is version 8, dated 24/02/2021.

## 3 Discussion

The APACAN2 trial aims to assess the feasibility of a home-based adapted physical activity program during neoadjuvant chemotherapy for HER2-positive breast cancer. In to this intervention, we aim to encourage physical activity from the beginning of the treatment by proposing a physical activity program at home and without time constraints. In addition, we focus on a population that can draw particular benefit from the practice of physical activity because of the treatment-induced cardiotoxity to which HER2-positive patients are exposed.

The current standard care to monitor cardiotoxicity is left ventricular ejection fraction (LVEF) monitoring (40–42). However, this method has some limits, as it lacks sensitivity, and does not enable early detection of cardiotoxicity. New techniques are currently developing, such as the titration of troponin in the blood. This biomarker is recognized as predictive of cardiotoxicity occurrence during treatment by the Food and Drug Administration and in several studies because it precedes decreases in LVEF (43–46). It would be interesting to follow levels of biomarkers such as troponin for the patients participating in the study.

We have chosen not to use a randomized design because it is a feasibility study and it was important that as many patients as possible should benefit from the intervention. According to the APACAN2 results, we could subsequently propose a randomized study. Another limit of our study is that the anthropometric measures and lipid test are done when the patient come for her consultation so they are not always fasting.

The strength of our study is that the physical activity program is proposed at the beginning of the treatment, as recommended. In the literature, the majority of studies deploy the physical activity program in the course of adjuvant chemotherapy or later. There is little data about physical activity and sedentariness during neoadjuvant chemotherapy for breast cancer. Furthermore, the home-based concept makes it possible to take geographical and temporal constraints into account. Indeed, patients are free to organise their sessions as they wish without complying with a specific imposed calendar, and they are not obliged to travel to a given place to train.

## Ethics Statement

The study protocol and its amendments has obtained approval from the French Ethics Committee (Comité de Protection des Personnes Sud-Est VI) (N°ID RCB 2016-A01344-47) in November 2016. The study is conducted in accordance with the Helsinki Declaration, the Good Clinical Practice (GCP) guidelines of the International Conference on Harmonisation (ICH–E6, 17/07/96).

## Author Contributions

AG, IM, ET and XD wrote the protocol. XD is the coordinator of the study. IM is the statistical expert, contributed to sample size calculations and developed the experimental plan. She will undertake the statistical analyses. MD supervised the physical activity evaluation. AG is the project manager of the study and is involved in aspects of the day-to-day running of the trial. She wrote the first draft of this manuscript and contributed to the grant proposal. All authors critically revised the manuscript, gave final approval of the manuscript and are accountable for the accuracy and integrity of the manuscript.

## Funding

This work received funding from the Quality of life Pink Ribbon Award by the association “Ruban Rose” in 2019 (www.cancerdusein.org).

## Conflict of Interest

The authors declare that the research was conducted in the absence of any commercial or financial relationships that could be construed as a potential conflict of interest.

## Publisher’s Note

All claims expressed in this article are solely those of the authors and do not necessarily represent those of their affiliated organizations, or those of the publisher, the editors and the reviewers. Any product that may be evaluated in this article, or claim that may be made by its manufacturer, is not guaranteed or endorsed by the publisher.

## Abbreviations

APA, Adapted physical activity; CI, confidence interval; HDL, High density lipoprotein; HER2, Human epidermal growth factor receptor 2; LDL, Low density lipoprotein; LVEF, Left ventricular ejection fraction; MFI-20, Multidimensional fatigue inventory; MSE, Medico-sports educator; NACT, Neoadjuvant chemotherapy; QLQ-C30, Quality of life questionnaire core 30; QOL; Quality of life; RPAQ, Recent physical activity questionnaire.
